# Supplementary material for: Geostatistical analysis to guide treatment decisions for soil-transmitted helminthiasis control in Uganda
Source: PLoS Negl Trop Dis. 2025 Sep 8;19(9):e0013467. doi: 10.1371/journal.pntd.0013467 (PMC12453197; doi:10.1371/journal.pntd.0013467)
Supplement: S2 Appendix — Fig A. These maps display the observed and predicted values of the proportion of households with improved sanitation (A and D), improved drinking water (B and E) and open defecation (C and F). These maps were created using the tmap-package in R and the shapefiles were downloaded from https://data.humdata.org/dataset/cod-ab-uga. Fig B. The geographical distribution of hookworm prevalence by treatment coverage by district. This map was created using the tmap-package in R, and the shapefile was downloaded from ESPEN (2022) - https://espen.afro.who.int/maps-data/data-query-tools/cartography-database (DOCX) [file pntd.0013467.s002.docx]

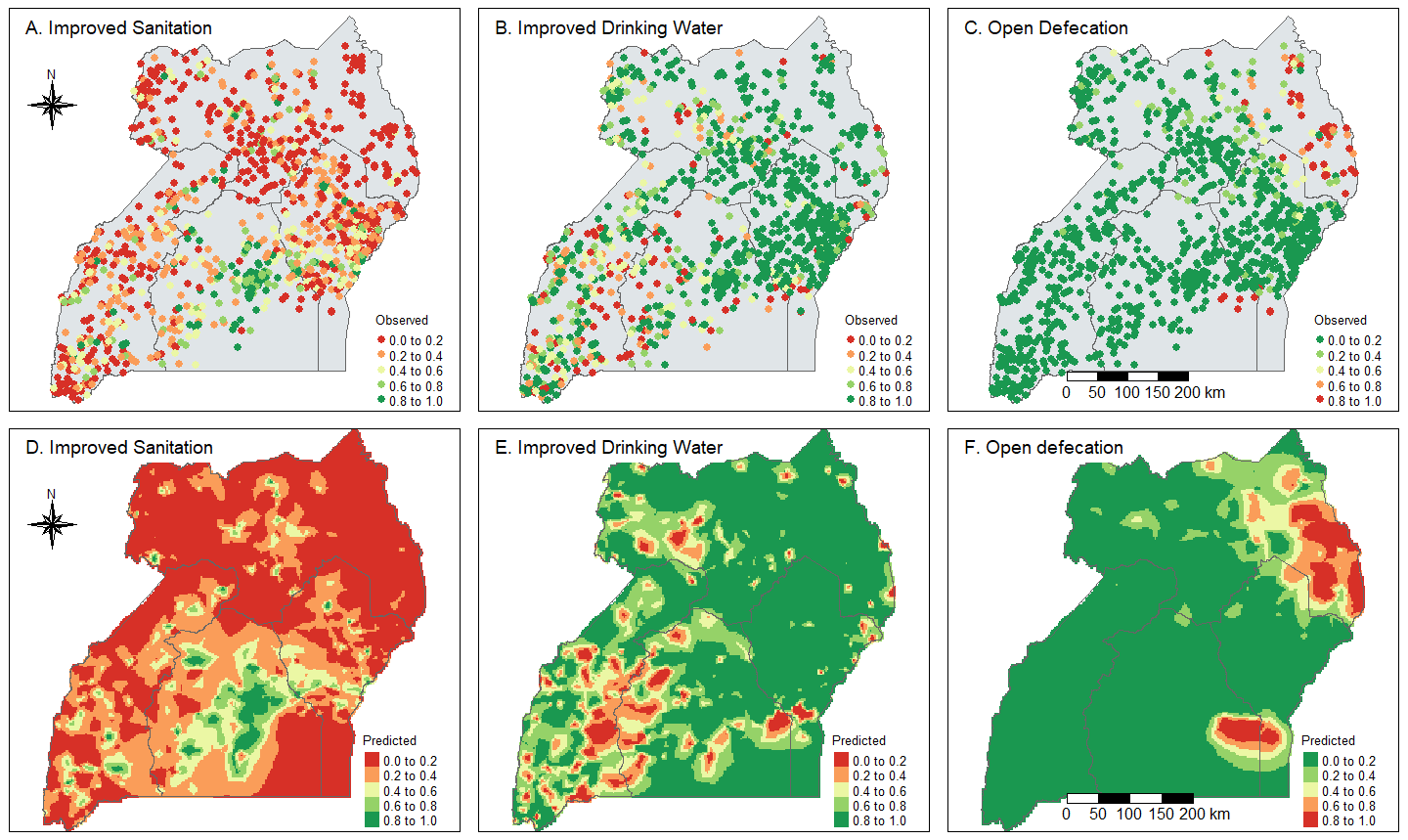


**Fig A:** These maps display the observed and predicted values of the proportion of households with improved sanitation (A and D), improved drinking water (B and E) and open defecation (C and F). These maps were created using the tmap-package in R and the basemap shapefiles were downloaded from <https://data.humdata.org/dataset/cod-ab-uga>


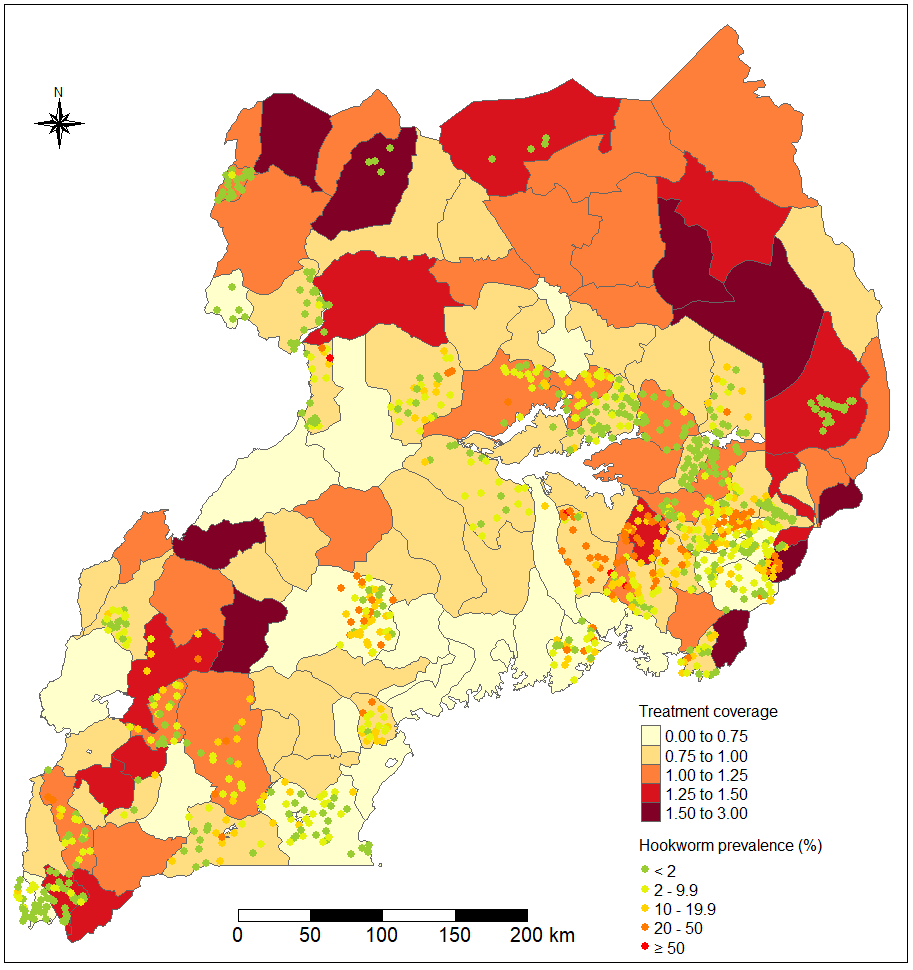


**Fig B:** The geographical distribution of hookworm prevalence by treatment coverage by district. This map was created using the tmap-package in R, and the basemap shapefile was downloaded from ESPEN (2022). <https://admin.espen.afro.who.int/docs/api/cartographies>
