# Supplementary material for: Geostatistical analysis to guide treatment decisions for soil-transmitted helminthiasis control in Uganda
Source: PLoS Negl Trop Dis. 2025 Sep 8;19(9):e0013467. doi: 10.1371/journal.pntd.0013467 (PMC12453197; doi:10.1371/journal.pntd.0013467)
Supplement: S3 Appendix — (DOCX) [file pntd.0013467.s003.docx]

**S3 Appendix: Bayesian model formulation**

Let $Y_{i}$ be the number of children who tested positive, $N_{i}$ be the total number of children tested, and $p_{i}$be the prevalence of infection in cluster $s_{i}$. We assumed that $Y_{i}$ comes from a binomial distribution $Y_{i}\sim Bin(N_{i} ,p_{i} )$ where $p_{i}$ is the probability of infection at cluster $s_{i}.$ The bivariate and multivariable binomial models were specified as:

$$logit\left( {p_{i}} \right)=\beta^{T}X\left( s_{i} \right)+\omega\left( s_{i} \right)+v(s_{i})$$

where $\boldsymbol{X}\left( s_{i} \right)$ is the set of predictors at location$s_{i}$, $\boldsymbol{\beta}$ = ($\beta_{1}, \beta_{2},\ldots,\beta_{k}$)^T^ is the vector of regression coefficients, $\omega$are the spatial random effects included in the model via the Matern covariance function and $v$represents the non-spatial random effect.

Given the excess zeros observed for *A. lumbricoides* (78% of the locations) and *T. trichiura* (86% of the location), the binomial distribution may not have been able to estimate the zero prevalence probability. We therefore fitted zero inflated binomial (ZIB) models $Y_{i}\sim ZIB\left( N_{i} ,p_{i},\theta_{i} \right),$ which assume two sources of zeros: $\theta_{i}$% (mixing probabilities) of structural zeros and $\left( {1-\theta}_{i} \right)\%$ arising from the binomial distribution. The model was defined as:

$$Y_{i}|p_{i},\theta_{i}\sim\left\{ \begin{aligned} 0 with probability \theta_{i} \\ Bin\left( N_{i} ,p_{i} \right) with probability 1-\theta_{i} \end{aligned} \right.$$

where the probability $\theta_{i}$ is modelled using predictors and was defined as ${logit(\theta}_{i})=\sum_{k=1}^{m} \alpha^{T}\boldsymbol{X}\left( s_{i} \right)$ where $\boldsymbol{X}\left( s_{i} \right)$ are covariates at location$s s_{i}$ and $\boldsymbol{\alpha}$ = ($\alpha_{1}, \alpha_{2},\ldots,\alpha_{k}$)^T^ are the regression coefficients. The relationship between $p_{i}$ and the predictors in ZIB models were modelled via the logit equation for $p_{i}$equation defined above.

We specified prior distributions for all model parameters. Specifically, we used vague normal priors for the regression coefficients $\beta\sim N(0, 1000),$ an inverse-gamma prior for the non-spatial variance$- \sigma_{non-spatial}^{2} \sim IG(2.01, 1.01)$, and penalized complexity priors for both the spatial variance and range, such that $P(\sigma_{\mathrm{spatial}}^{2} > 5) = 0.01$ and $P(r < 111.13/4) = 0.05$. To account for excess zeros, we employed a zero-inflated binomial model with covariates in the zero-inflation component, using the "0binomial" family in INLA. This formulation allows the probability of structural zeros to be modeled through a latent linear predictor, incorporating covariate effects. Priors for the zero-inflation coefficients were specified through the “control.family” argument as $\beta\sim N(0, 1)$.
