# Supplementary material for: Geostatistical analysis to guide treatment decisions for soil-transmitted helminthiasis control in Uganda
Source: PLoS Negl Trop Dis. 2025 Sep 8;19(9):e0013467. doi: 10.1371/journal.pntd.0013467 (PMC12453197; doi:10.1371/journal.pntd.0013467)
Supplement: Attachment — Submitted filename: Response to Reviewers_20250703.docx [file pntd.0013467.s004.docx]

**gPoint-by-point response to reviewers’ comments for manuscript number: PNTD-D-25-00325**

We are very thankful to both the reviewers and the editors for their invaluable comments and suggestions which have improved our manuscript. Below, you’ll find our point-by-point response to the reviewers’ comments, clearly indicating the changes made throughout the manuscript. Please note that in this response we highlight the line numbers from the tracked version of the manuscript.

**Editors**

**Journal Requirements:**

1) Please ensure that the CRediT author contributions listed for every co-author are completed accurately and in full.
At this stage, the following Authors/Authors require contributions: Bryan O. Nyawanda, Kristin M. Sullivan, Benjamin Tinkitina, Prudence Beinamaryo, Betty Nabatte, Hilda Kyarisiima, Alfred Mubangizi, Paul M. Emerson, Jürg Utzinger, and Penelope Vounatsou. Please ensure that the full contributions of each author are acknowledged in the "Add/Edit/Remove Authors" section of our submission form.
The list of CRediT author contributions may be found here: [https://journals.plos.org/plosntds/s/authorship#loc-author-contributions](https://scanmail.trustwave.com/?c=22737&d=-uqg6KxyQ9QLFkcb51LG4GgZ665poMik4UlVbw8F6Q&u=https%3a%2f%2fjournals%2eplos%2eorg%2fplosntds%2fs%2fauthorship%23loc-author-contributions)

Thank you for highlighting this. We included the requested author contributions in the manuscript, but we will edit this section of the submission form as appropriate.

2) Please upload all main figures as separate Figure files in .tif or .eps format. For more information about how to convert and format your figure files please see our guidelines: 
[https://journals.plos.org/plosntds/s/figures](https://scanmail.trustwave.com/?c=22737&d=-uqg6KxyQ9QLFkcb51LG4GgZ665poMik4UgKb1gO7g&u=https%3a%2f%2fjournals%2eplos%2eorg%2fplosntds%2fs%2ffigures)

We have now prepared the figures in .tif format ready for upload to the Editorial Manager. Many thanks.

3) Some material included in your submission may be copyrighted. According to PLOSu2019s copyright policy, authors who use figures or other material (e.g., graphics, clipart, maps) from another author or copyright holder must demonstrate or obtain permission to publish this material under the Creative Commons Attribution 4.0 International (CC BY 4.0) License used by PLOS journals. Please closely review the details of PLOSu2019s copyright requirements here: PLOS Licenses and Copyright. If you need to request permissions from a copyright holder, you may use PLOS's Copyright Content Permission form.
Please respond directly to this email and provide any known details concerning your material's license terms and permissions required for reuse, even if you have not yet obtained copyright permissions or are unsure of your material's copyright compatibility. Once you have responded and addressed all other outstanding technical requirements, you may resubmit your manuscript within Editorial Manager. 
Potential Copyright Issues:
i) Figures 1, 2, 3, and S2. Please (a) provide a direct link to the base layer of the map (i.e., the country or region border shape) and ensure this is also included in the figure legend; and (b) provide a link to the terms of use / license information for the base layer image or shapefile. We cannot publish proprietary or copyrighted maps (e.g. Google Maps, Mapquest) and the terms of use for your map base layer must be compatible with our CC BY 4.0 license.
Note: if you created the map in a software program like R or ArcGIS, please locate and indicate the source of the basemap shapefile onto which data has been plotted.
If your map was obtained from a copyrighted source please amend the figure so that the base map used is from an openly available source. Alternatively, please provide explicit written permission from the copyright holder granting you the right to publish the material under our CC BY 4.0 license.
If you are unsure whether you can use a map or not, please do reach out and we will be able to help you. The following websites are good examples of where you can source open access or public domain maps:
* U.S. Geological Survey (USGS) - All maps are in the public domain. ([http://www.usgs.gov)](http://scanmail.trustwave.com/?c=22737&d=-uqg6KxyQ9QLFkcb51LG4GgZ665poMik4R0JOQoE7Q&u=http%3a%2f%2fwww%2eusgs%2egov%29)
* PlaniGlobe - All maps are published under a Creative Commons license so please cite u201cPlaniGlobe, [http://www.planiglobe.com,](http://scanmail.trustwave.com/?c=22737&d=-uqg6KxyQ9QLFkcb51LG4GgZ665poMik4RgPbwwNuA&u=http%3a%2f%2fwww%2eplaniglobe%2ecom) CC BY 2.0u201d in the image credit after the caption. ([http://www.planiglobe.com/?lang=enl)](http://scanmail.trustwave.com/?c=22737&d=-uqg6KxyQ9QLFkcb51LG4GgZ665poMik4RMOMglcvQ&u=http%3a%2f%2fwww%2eplaniglobe%2ecom%2f%3flang%3denl%29)
* Natural Earth - All maps are public domain. ([http://www.naturalearthdata.com/about/terms-of-use/).](http://scanmail.trustwave.com/?c=22737&d=-uqg6KxyQ9QLFkcb51LG4GgZ665poMik4UhbagsPuA&u=http%3a%2f%2fwww%2enaturalearthdata%2ecom%2fabout%2fterms-of-use%2f%29)

Thank you for highlighting this. The basemaps we have used are open access and are provided by the World Health Organization’s Expanded Special Project for the Elimination of Neglected Tropical Diseases (ESPEN) - <https://espen.afro.who.int/maps-data/data-query-tools/cartography-database> and the United Nations’ Office for the Coordination of Humanitarian Affairs <https://data.humdata.org/dataset/cod-ab-uga>. We have edited the Figure captions adding the reference numbers to the urls. These maps were then processed in R using the tmap package.

4) Please amend your detailed Financial Disclosure statement. This is published with the article. It must therefore be completed in full sentences and contain the exact wording you wish to be published. Please ensure that the funders and grant numbers match between the Financial Disclosure field and the Funding Information tab in your submission form. Note that the funders must be provided in the same order in both places as well. State the initials, alongside each funding source, of each author to receive each grant. For example: "This work was supported by the National Institutes of Health (####### to AM; ###### to CJ) and the National Science Foundation (###### to AM).".
If you did not receive any funding for this study, please simply state: u201cThe authors received no specific funding for this work.u201d

We have specified the funding statement within the manuscript. Thank you.

**Reviewer: 1**

**General**

The authors should be commended on a well thought-out and executed analysis. Their application of geostatistical modelling to guide programmatic decision making is informative. Please find in the relevant sections comments for consideration
**Response:** We thank Reviewer #1 for the positive appraisal of our research.

**Methods**

The study design and statistical methods are largely appropriate to address the objectives of the analysis and there are not concerns about ethical or regulatory requirements. Some additional comments for consideration:

**Response:** Thank you for your feedback.

1. Page 7, line 131 - it would be good to know how many studies incorporated WASH and treatment data, and whether this was individual-level data or aggregate data.

**Response:** Thank you for this helpful point. We use data from three surveys described in lines 134-139. These surveys did not conduct the kind of analysis presented in our manuscript. In our work, we combine the survey data with other data and conduct a geostatistical analysis to align WASH with STH survey locations as outlined in lines 140-169. In response to your comment, we have added clarification on the aggregation level of the WASH indicators in lines 155-156 and for treatment data in line 159. We also considered the potential influence of Lymphatic Filariasis treatment on STH infection prevalence and included text to highlight this consideration in lines 159-163. We have also specified in lines 166-167 that all data were processed at the survey location level.

2. Page 9, lines 181-184 - please clarify why the authors approach overall STH infection in this manner (equation based on individual STH species) as opposed to using empiric overall STH infection data (similar to how species-specific data is used). This analysis is focused on programmatic decision making

**Response:** Thank you for this question. Our analysis was based on secondary data, and empirical prevalence of any STH infection were available only for a subset of survey locations. Furthermore, geostatistical modelling was conducted at the species level, as climatic and environmental drivers differ between STH species. As a result, we adopted a probabilistic approach to estimate overall STH prevalence from species-specific estimates. Recognizing the limitations of this approach, we adjusted the estimates using correction factors, proposed by Silva *et al.* (2010). Please see lines 205-208 for the additional text explaining the adjustment process and lines 438-440 for additional text on the potential difference in prevalence arising from predictors.

3. Page 9, lines 187-191 - please include any precedence of references for this approach.

**Response:** Posterior samples are drawn and used in the estimation. The approach we applied to assign pixels to endemicity classes has been previously used by Gerber *et al.* (2023) (see: <https://journals.plos.org/plosntds/article?id=10.1371/journal.pntd.0011656>), although the method was not explicitly described in their manuscript. A more detailed application of the approach can be found in Nyawanda *et al.* (2024), where it is clearly defined for mapping changes in malaria risk (see: <https://ij-healthgeographics.biomedcentral.com/articles/10.1186/s12942-024-00381-8>). Both references have now been included in the revised manuscript.

4. Page 9, line 197 - what about treatment frequency for STH prevalence >50%?

**Response:** Thank you for this point. For the classification into treatment categories, we followed the most recent WHO guidelines, in which the >50% prevalence class has been dropped.

5. Page 9, lines 202-204 - could not this mapping be the same as page 9 lines 184-187?

**Response:** We have revised and restructured the sentences in lines 228-231 to improve clarity. We hope this now clearly distinguishes the two approaches.

6. Page 10, line 216 - please specify what treatment categories 1-4 mean.

**Response:** Thank you for this suggestion. We have now specified the definitions of treatment categories 1-4 in line 244 as recommended.

7. Please include the methods for producing the uncertainty estimates and maps.

**Response:** We have added explanatory text in lines 198-200 regarding the generation of the uncertainty map, and in lines 244-247 for the prevalence estimates.

**Results**

Overall the results are well presented and the figures are of sufficient quality. Some additional comments for consideration:

**Response:** Thank you for this feedback.

1. Page 10, line 228 - can the authors include a descriptive estimate for any STH infection?

**Response:**  We have now included the observed prevalence estimates for each species and for any STH infection in lines 260-262.

2. Table 1 - it may be good for readers to understand why the rows do not necessarily add up to the overall models.

**Response:**  We have included a clarifying note below the table.

3. Table 1 - the authors could provide some insights as to their thoughts on why there is such a discrepancy in treatment as a predictor across the species.

**Response:**  Indeed, this was an interesting finding. We have included our interpretation and potential explanations in the third paragraph of the discussion section.

4 Page 13 - could a model be developed for overall STH infection (in keeping with comment 2 for methods)?

**Response:**  Thank you for this point. Unfortunately, it was not possible to develop such a model because individual-level data were available for a subset of survey locations. Hence, we modelled species-specific prevalence and then estimated the prevalence of any STH infection. To account for overestimation, we applied the correction proposed by Silva *et al.* (2010) (see <https://journals.plos.org/plosntds/article?id=10.1371/journal.pntd.0000655>).

5. Page 17, lines 319-321 - please clarify how these national predicted estimates were derived (e.g., in methods).

**Response:**  Thank you for pointing this out. We have added explanatory text in lines 244-247 of the Methods section to clarify how the national estimates were derived.

**Conclusion**

The conclusions are supported and limitations are clearly described. Additional things to consider:
1. It would be good to have more understanding of the current Ugandan STH control program, this can be in the introduction. Drug requirement is only brought to attention in the discussion. Given a major focus is on how this analysis can optimise drug procurement the manuscript would benefit by providing more background to the Ugandan STH control program in the introduction.

**Response:**  We appreciate this comment. In response, we have added text in lines 97-98 to strengthen the Background section and provide details on the current preventive chemotherapy distribution practises in Uganda.

2. Page 21, lines 409-412 - the authors could elaborate more on how geostatistical modelling can support drug procurement at smaller spatial scales.

**Response:**  Thank you for this observation. Geostatistical modelling revealed substantial within district variation, which supports our recommendation to consider drug procurement at finer spatial scales. Since our predictions are made at 1 x 1 km^2^ resolution, aggregation can be adapted as needed by the control program to inform more targeted decision making. Please see lines 471-474.

**Reviewer: 2**

This paper adds to the evidence that geostatistical methods can enable more focused application of preventive chemotherapy, thus reducing the total cost of an effective control strategy. I have concerns about some aspects of the methodology and/or its exposition that I think need to be addressed before publication.

**Response:** We thank Reviewer #2 you for the feedback.

Specific
1. The authors acknowledge that their approach to model selection is “non-conventional.” I agree, which makes it all the more important that it be described clearly to allow others to emulate it. I found Table 1 hard to understand. The test states that “The relative frequency with which each predictor was included in the top models with similar WAIC is summarized in Table 1.” I therefore expected each set of three integers in each row of Table 1 to add to the number of models under consideration, which is clearly not the case. What am I missing?

**Response:** Thank you for this observation. The top-ranked models contain different combinations of predictors, so not all variables are included in every model. Hence, the three numbers per species in each row of Table 1 represent counts from only the subset of top models in which that variable was included, not from the total number of models under consideration. For example, in the case of organic carbon stock for *Ascaris lumbricoides*, the variable was included in 43 out of the 71 top models. Among those 43 models, it was found to be not statistically important in 27 and positively associated in 16. We have added a note below the table to clarify this for readers.

2. I am sceptical of completely automated variable selection methods. In my opinion, contextual knowledge should also play a role, to distinguish between covariates for which there is or is not an a priori expectation of their importance. The authors should at least comment, in the discussion section, on how their preferred models do or do not have face validity in this sense.

**Response:** Thank you for this point. In previous work, we have incorporated contextual knowledge into the variable selection process, as described by Karagiannis-Voules *et al.* (2015) (see: <https://www.thelancet.com/journals/laninf/article/PIIS1473-3099(14)71004-7/fulltext>). However, in the current study, our aim was to identify the model with the best predictive ability to inform the classification of districts into treatment categories. It is also worth noting that biologically important variables may not have strong statistical signal in a dataset due to low variation or confounding. In light of the study’s goal, we adopted a prediction-focused methodology and discussed the approach, including limitations, in lines 476-490.

3. I assume that the authors used the sample functionality within the INLA software, as this is essential for the validity of area-level probability statements. If so, this should be stated explicitly.

**Response:** We have now clarified in lines 250-251 of the Methods section that we used the sample functionality in INLA to support the generation of area-level probability statements.

4. The assumption that the three specific prevalences are independent is dubious. What evidence (empirical or otherwise) is there to support this? How different would the results be if the “any STH” response was defined empirically.

**Response:**  Thank you for this important point. Ideally, the prevalence of any STH infection should be defined empirically using individual-level data. However, in this analysis, individual-level data were available for only a subset of survey locations. Given this limitation, we used a probabilistic approach to estimate the prevalence of any STH from species-specific estimates. We believe that the method used in estimating the prevalence of any STH is robust as it has been used in several studies. We acknowledge the limitations of the independence assumption. To address this, and in response to this and similar comments from other reviewers, we applied a correction based on the findings of Silva *et al.* (2010) (see: <https://journals.plos.org/plosntds/article?id=10.1371/journal.pntd.0000655>). We believe the method used is robust and consistent with approaches adopted in previous studies, including: Khaki et al. (2025) (see: <https://pmc.ncbi.nlm.nih.gov/articles/PMC11753640/>); Gerber *et al.* (2023) (see:<https://journals.plos.org/plosntds/article?id=10.1371/journal.pntd.0011656>) and Lai *et al.* (2019) (see: <https://journals.plos.org/plosntds/article?id=10.1371/journal.pntd.0007580>). We have updated the text accordingly to clarify this approach and acknowledge its limitations.

5. The counter-intuitive finding that “increased treatment was positively significantly associated with STH infection” suggests strongly that the sampled locations were biased in favour of high-risk areas. The authors should describe what sampling designs (if any) were used and what are the implications for their findings. Incidentally, the use of “significantly” conflicts with their earlier statement about avoiding the use of this word having no place in Bayesian inference.

**Response:** Thank you for these helpful comments. We discuss the counter-intuitive finding in lines 423-435 of the Discussion. We acknowledge that this association may be partly explained by sampling bias, as surveyed locations may have been selected based on known high-risk areas. To address this, we suggest that additional surveys could help resolve this issue. Furthermore, we note that other socio-behavioural factors may also play a role and could be better understood through targeted studies. We also appreciate your point regarding the use of the term “significantly.” We have now revised the manuscript to avoid this term and ensure consistency with Bayesian inference principles throughout.

6. I could find no mention of prior distributions - these should be stated and justified. Also, for the preferred model, priors should be compared with posteriors to check that the inferences are not being driven by the priors.

**Response:** Thank you for pointing this out. We have now added a description of the prior distributions used in the model formulation in S3 Appendix, along with justification. We also acknowledge the importance of comparing prior and posterior distributions and as such, we have included text in the Results section highlighting this comparison in the revised manuscript (lines 304-306).

7. Nor could I find any diagnostic checks on the preferred model.

**Response:** Thank you for this comment. We applied different approaches to select and evaluate the final model. First, we used the Watanabe-Akaike Information Criterion (WAIC) to identify the top-performing models. WAIC evaluates predictive accuracy using the full posterior distribution and balances goodness of fit with model complexity to reduce the risk of overfitting. Second, we applied a leave-one-out cross-validation method to assess predictive performance by comparing predicted and observed prevalence values. We report the root mean square error (RMSE) and mean absolute error (MAE) as performance metrics, as described in the statistical analysis section (lines 172-201).

8. An additional reference to the use of geostatistical models to enable more focused application of PC against STH is: Ediriweera et al (2019). Reassessment of the prevalence of soil-transmitted helminth infections in Sri Lanka to enable a more focused control programme: a cross-sectional national school survey with spatial modelling. Lancet Global Health, [http://dx.doi.org/10.1016/S2214-109X(19)30253-0.](http://scanmail.trustwave.com/?c=22737&d=-uqg6KxyQ9QLFkcb51LG4GgZ665poMik4UkIPwheug&u=http%3a%2f%2fdx%2edoi%2eorg%2f10%2e1016%2fS2214-109X%2819%2930253-0)

**Response:** We appreciate this valuable source and provide cross-reference in our manuscript.

**Reviewer: 3**
**General**

Reviewer #3: Authors provide a detailed and thoughtful use of geostatistics to identify optimal predictors of different STH species, and classiy sub-national areas of Uganda into policy relveant prevalnce thresholds. The article is well written, albeit perhaps a little too technical for the general readership of PLoS NTDs. The analysis is well performed and results are presented clearly . I have a few minor comments/clarifications which may strengthen the manuscript:

**Response:** We thank Reviewer #3 for the overall positive appraisal of our work.

- In the intro, it might be good to mention that geostatistics is one of the recommended methodologies in the new WHO STH M&E guidelines. It has also been used in a couple of other settings to achieve similar goals (Kenya, Ethiopia), which authors could mention.

**Response:** Thank you for this helpful suggestion. While we briefly alluded to the use of geostatistical methods in paragraph 2 of the Introduction, we had not stated it explicitly. We have now revised the text in lines 92–93 to clearly mention that geostatistical modelling is recommended in the latest WHO STH M&E guidelines and has been applied in other settings, such as Kenya and Ethiopia.

- The species specific point estimates presented in the abstract, the total of the 3 species is quite far form the any STH. I imagine this is due to seperate optimal covariate models being fit for each, but it will be confusing for non-technical audiences. I suggest providing an explanation in the body of the text, and consider removing these figures from the abstract - perhaps describing the overall species specific, and any STH, probability bands instead?

**Response:** Thank you for this comment. The discrepancy arises from the method used to generate the summary statistics, which accounts for heterogeneity. To improve clarity, we have revised how the statistics are presented and have updated both the Abstract and the Results section in the main text.

- The results have the requiste pills needed ina a given year to demonstrate the efficiency of sub-national targeting, but WHO guidelines work on a 2-year basis, could authors correct these calculations to show the 2 year tield.

**Response:** Thank you for this comment. We choose to present annual estimates to make the findings easier for national control programs to interpret and communicate with stakeholders. These figures can be readily scaled to a two-year treatment cycle as needed. Internally, program managers can apply the appropriate treatment intervals based on WHO guidelines.

- The analysis utilised data from 2 survey types: community and school. Was there any attempt to account for the likely variance between these two data sources (Community likely incorproate out of school children which means school based estimates may be artifically low).

**Response:** Thank you for highlighting this important point. We agree that school-based surveys may underestimate STH prevalence due to the exclusion of out-of-school children, who are more likely to be captured in community surveys. Unfortunately, the community-based survey did not collect school attendance data, which limited our ability to quantify this potential bias directly. However, we recognize this as a limitation of the study and have acknowledged the potential for systematic differences between the two data sources in the revised manuscript (lines 494-498).

- The validatoin of the different species specific models used an 85%/15% split of training vs. test data. Was there any basis for using this split?

**Response:** Yes, this choice was informed by the data at hand. We selected an 85%/15% training-to-test split in order to maximize the data used for model training, while still retaining enough observations for testing model performance. We have added text explaining this rationale in lines 192-193.

- Table 1, the total number of models tested appears different for each species. Why was this?

**Response:**  The total number of models differ by species because we only retained the set of top-performing models for each species, defined as those with the lowest WAIC values within 4 units of the best model. This threshold ensures that only similarly performing models are considered, and the number of such models varies by species. This approach is described in the Methods (lines 179-183) and Results (lines 275-287) sections. In response to this comment, we have added a clarifying note below Table 1 to help readers in interpreting the numbers across the rows.

- The approach of using a 10% level of probability seems novel, could the authors expand on this, namely why 10% was chosen. Also perhaps a couple of examples in text, for example, what would happen if probabilities were v_low 35%, low 35%, moderate 30%, high 0% or something like that. Though, perhaps I'm not understanding he classification algorithm correctly.

**Response:**  Thank you for raising this important point. The 10% threshold was originally based on the assumption of five WHO treatment categories, where the uniform probability of a district falling into any given category would be 20%. A difference of 10% between the highest and second-highest probabilities (i.e. 50% of 20%) was considered a reasonable cut-off to indicate increased classification certainty. Given that the classification has since been revised to four categories, the probability is now 25%, and half of this is 12.5%. We have updated the manuscript accordingly and included a clear explanation in lines 235-238.

Regarding the example, when the probabilities are 35%, 34%, 31% and 0%, the difference between the highest and second-highest probabilities is only 1%, which is well below the 12.5% threshold. In such cases, we conservatively assign the district to the second treatment category to avoid overconfidence in uncertain classifications. This logic is explained in lines 238-241 and further illustrated in the Results section (lines 343-352).

- It seems this article is jointly aiming to be a methods article, and also a policy discussion on updated MDA needs but also the use of geostats to improve efficiency. Could the authors add a bit more explanation on the latter, perhaps incorporate the table in the supplementary material which highlights the classification probabilities into the main text and spend a few sentences describing how this might be interpreted?

**Response:**  Thank you for this helpful comment. Our article is indeed intended to serve both methodological and policy-relevant purposes: to demonstrate how geostatistical approaches can support more efficient allocation of treatment needs, and to present the results in a way that is accessible to both technical and programmatic audiences. We aimed to ensure that analysts can understand and potentially replicate the methods, while epidemiologists and control program managers can interpret and apply the findings in policy contexts. In response to Reviewer #3’s suggestion, we have expanded the text in the Results section (lines 343-352) to explain how the classification probabilities presented in the supplementary table can be interpreted. We highlight how this probabilistic information can inform programmatic decision-making by showing the confidence associated with each district’s assigned treatment category.

**Methods**

Yes, and no concerns about ethical or regulatory requirements

**Response:** Thank you for your feedback.
